# Supplementary material for: Long range and highly tunable interaction between local spins coupled to a superconducting condensate
Source: Nat Commun. 2021 Nov 18;12:6722. doi: 10.1038/s41467-021-26802-x (PMC8602442; doi:10.1038/s41467-021-26802-x)
Supplement: Supplementary file 1 — Supplementary Information [file 41467_2021_26802_MOESM1_ESM.pdf]

Supplementary Information
Long range and highly tunable interaction between local spins coupled to a superconducting condensate

Küster *et al.*

October 15, 2021

Supplementary Note 1: Metallic vs. superconducting STM tip

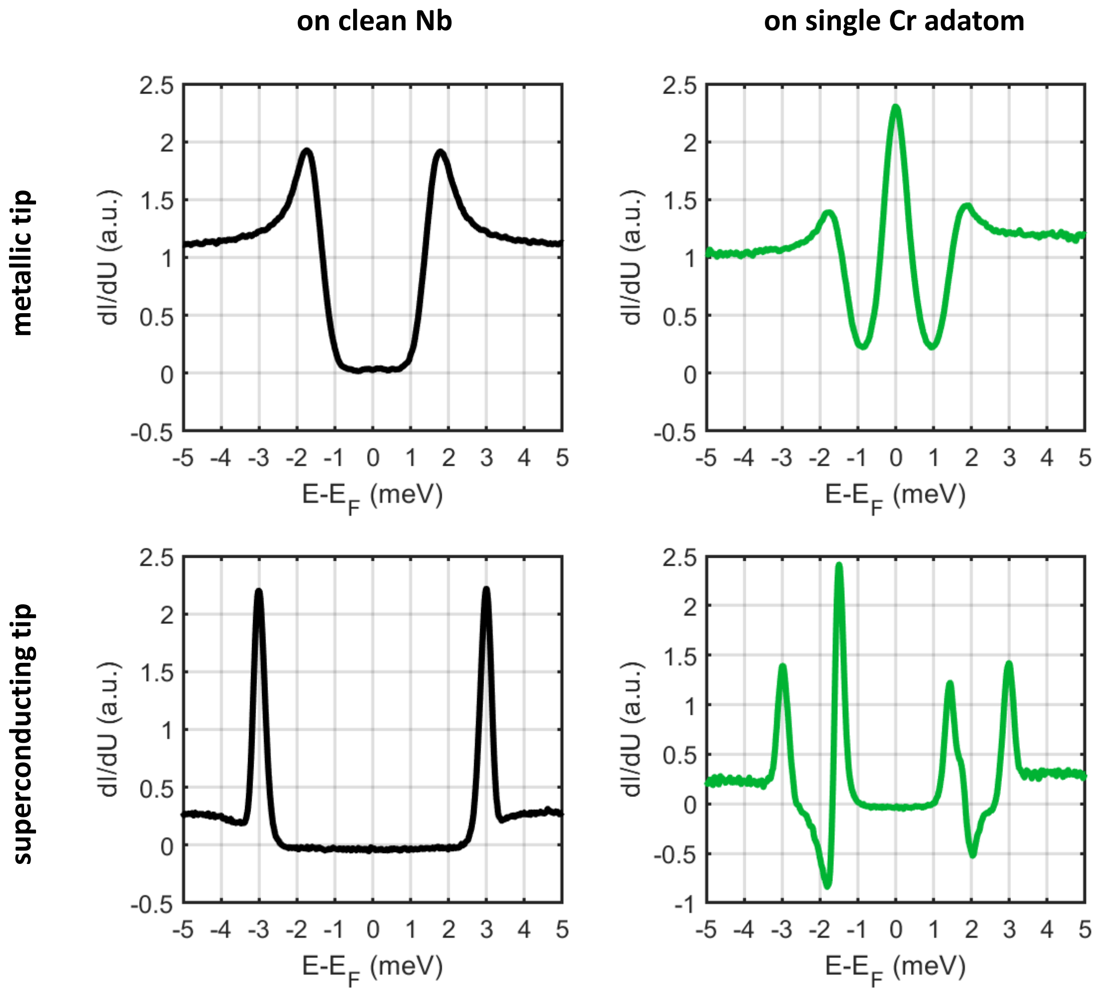

Supplementary Fig. 1: Enhancing energy resolution beyond Fermi-Dirac limit by using a superconducting probe: The figure shows two STS each taken on clean Nb (black) and on a single Cr adatom (green), one with a metallic STM tip (top) prepared on the Ag(111) surface and one with a superconducting tip (bottom) obtained by tip indentations to into the Nb single crystal. With the metallic tip, we observe a superconducting gap ($\Delta_{\mathrm{Nb}} = 1.53$meV), which becomes doubled with the superconducting Nb tip, featuring very sharp coherence peaks and a sub-meV energy resolution. On a single isolated Cr atom with a metallic tip, a YSR resonance at zero bias is observed, which shows a higher spectral weight on the negative side only when a superconducting tip is used that shifts all spectral features about $\pm\Delta_{\mathrm{tip}}$. Temperature during measurement: 0.55K.

Supplementary Note 2: Multi-orbital nature of YSR states

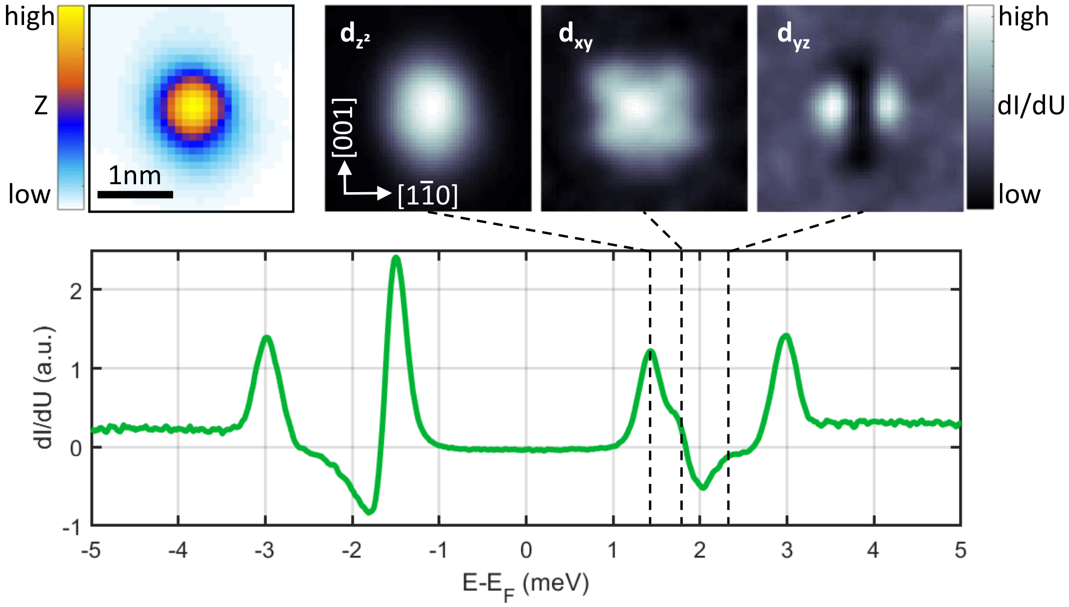

Supplementary Fig. 2: The figure shows spatial imaging of YSR resonances corresponding to different atomic orbitals. The topography data in the left panel has the same scale like the constant energy dI/dU maps to the right, taken at energies 1.50 meV, 1.77 meV and 2.28 meV, from left to the right, indicated with dashed lines in the spectroscopy curve below taken on a single Cr adatom. Using STS, we observe the peak that corresponds to the Cr d_{z^2} orbital as the most prominent one due to its longer extension into vacuum compared to the others. Additionally, it is the YSR state with the lowest energy, i.e. the peak is extremely close to Δ_{tip} which means zero energy, therefore offering high sensitivity for shifts of the YSR energy and the possibility to tune the system through a quantum phase transition more easily by interaction with other spins. The d_{xy} orbital is at higher energy, however, it partially overlaps in energy with the d_{z^2} peak. The d_{yz} orbital is energetically and spatially very separated from the other two, therefore it is not important in the analysis we did in this work.

Supplementary Note 3:   Mapping dimer states

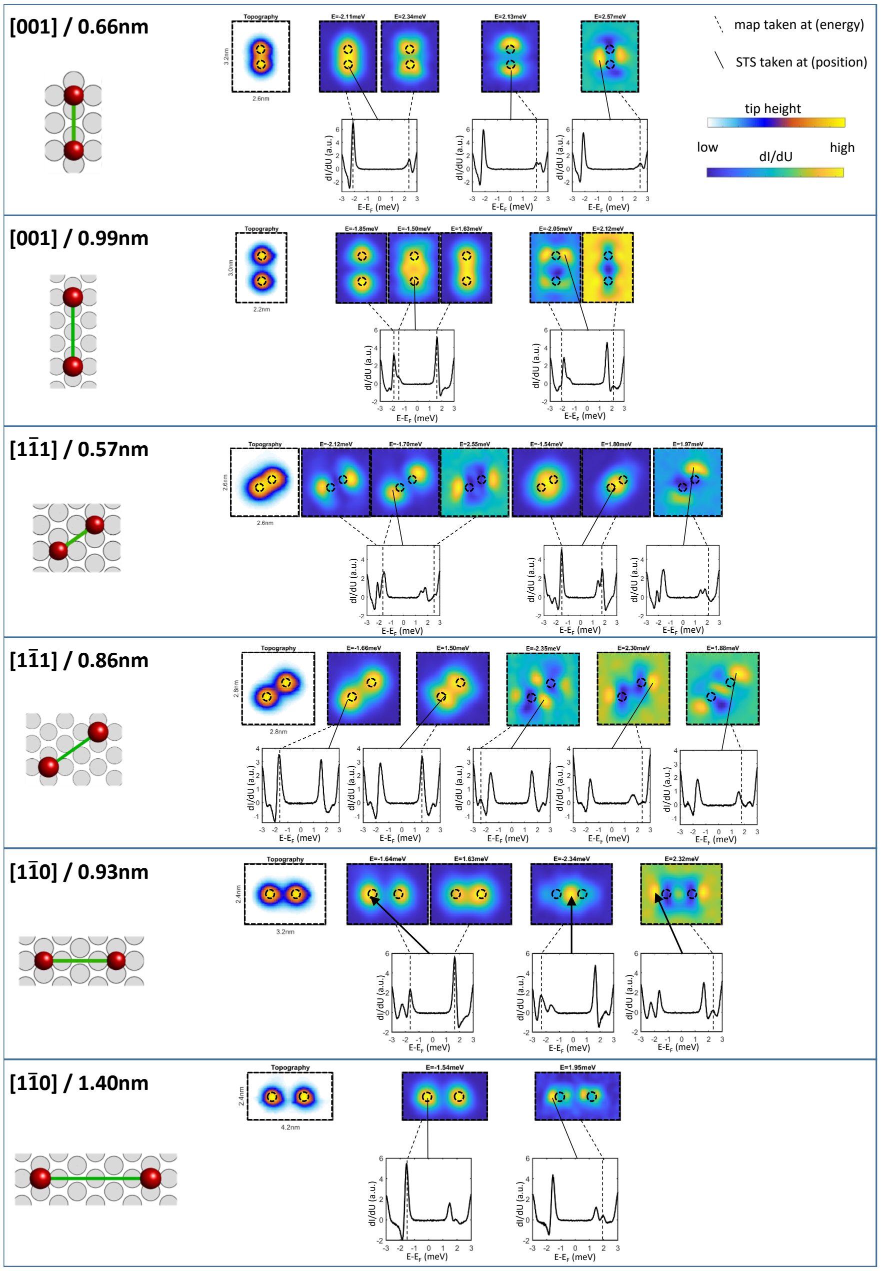

Supplementary Fig. 3: Detailed analysis of the first two distance steps of Cr dimers aligned along 3 distinct crystallographic directions: [001], [1$\bar{1}$1], and [1$\bar{1}$0]. For each dimer, dI/dU maps at the energies indicated in the STS spectra below (see black dashed lines) are reported. The STS spectra were taken with the tip in the position indicated by the continuous black lines. The emergence of hybridized YSR states characterized by a distinct spatial distribution compared to the isolated adatom case (reported in Figure S2) can be clearly revealed. Although at the limit of our experimental energy resolution, the formation of bonding and anti-bonding YSR states of different orbital origin can be also revealed in most of the cases.

Supplementary Note 4: The correlation between a small YSR energy shift and the in-gap peak intensities: a numerical simulation

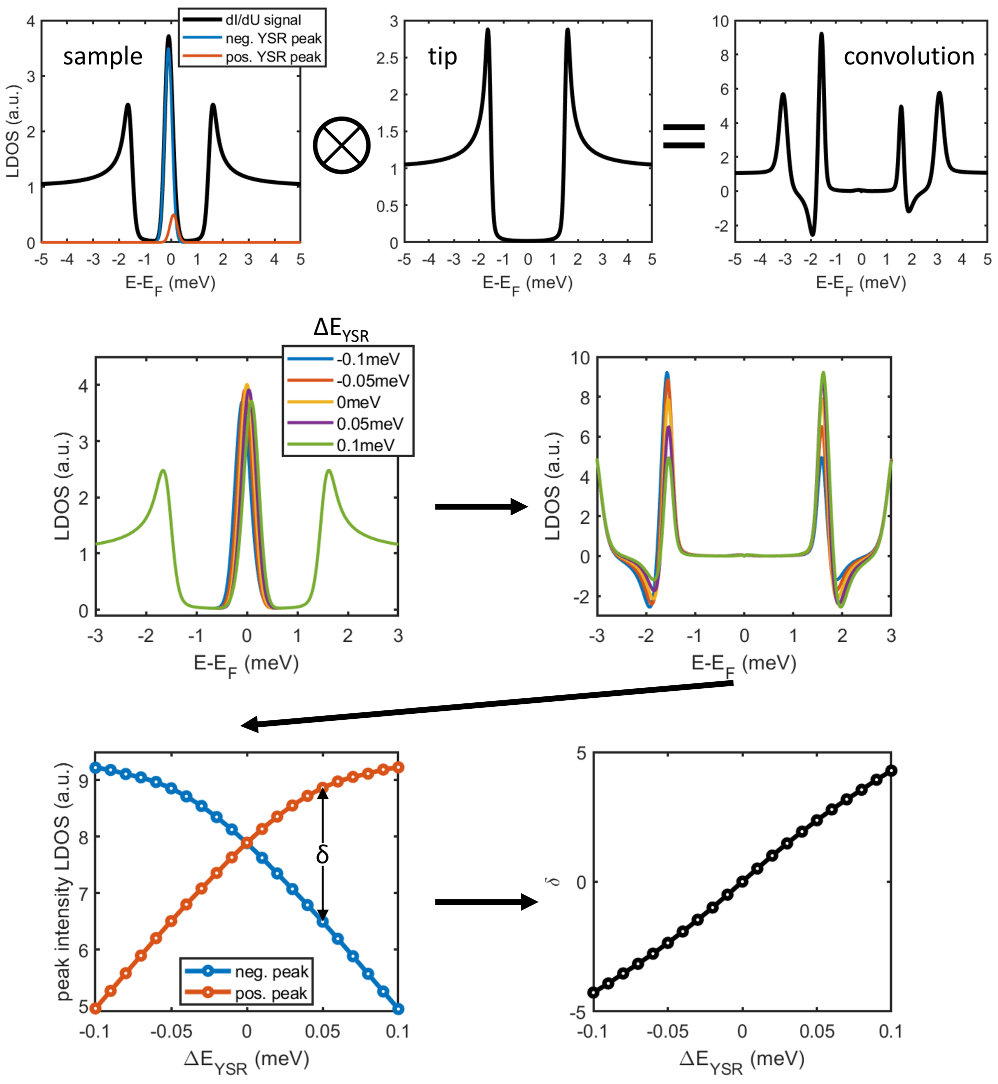

Supplementary Fig. 4: Numerical simulation of the correlation between a small shift in the YSR energy ΔE_{YSR} of $\pm 100\,\mathrm{meV}$ around zero and the analysis parameter δ which was used in this work. Top panels: A dynes function combined with two peaks symmetric around, but close to, zero energy, convoluted with another dynes function was used to describe the dI/dU signal measured during the experiment with a superconducting tip. Middle panels: A small shift of YSR energy, below the limit of our energy resolution, results in a significant change of the in-gap peak intensities in the convoluted spectrum, enabling the detection of tiny coupling effects in the experiment. Bottom panels: The analysis parameters P_+, P_- (positive and negative peak intensity) and $\delta = P_+ - P_-$. In a small energy window around zero, there is approximately a linear correlation between δ and ΔE_{YSR}.

Supplementary Note 5: Raw dI/dU signals from distance dependence measurements

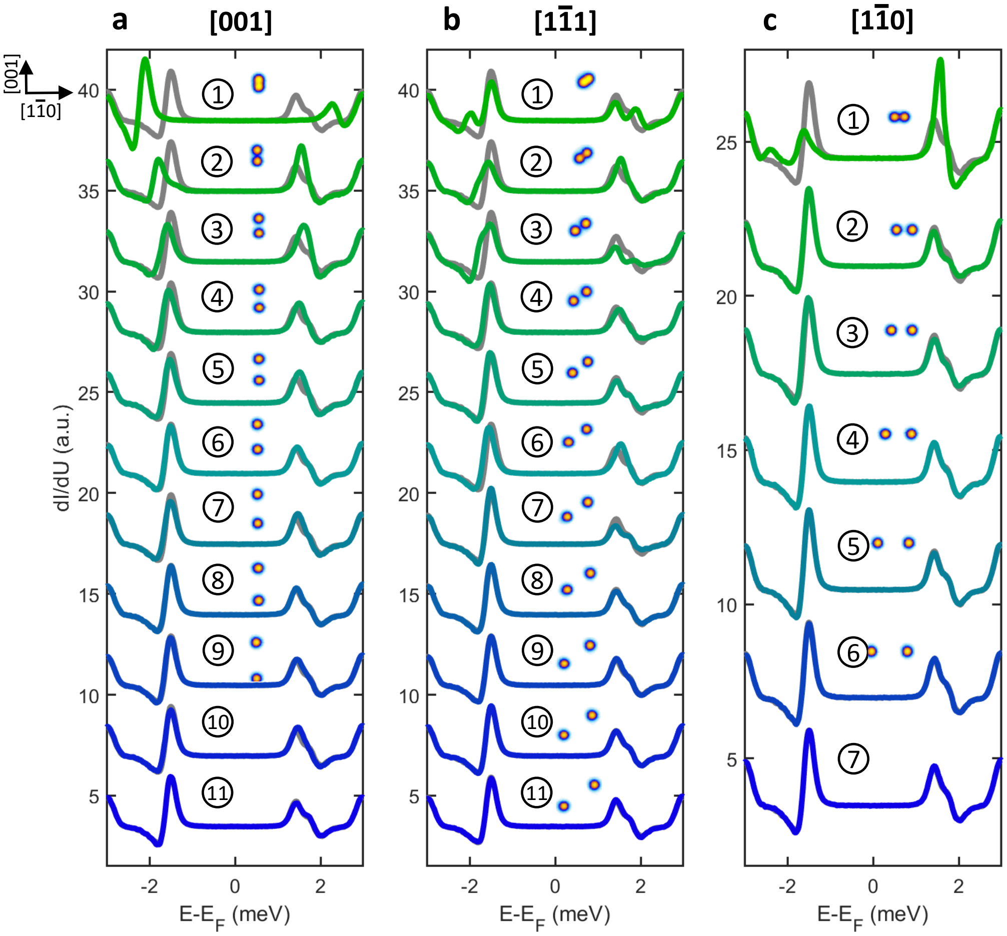

Supplementary Fig. 5: Distance dependence of the indirect coupling between two magnetic Cr adatoms along three directions: Raw dI/dU data taken on the fixed Cr of the adatom pair overlapped with the single, isolated Cr reference (gray) and vertically displaced for visibility. Step 1 corresponds to the closest separation, i.e. 0.66 nm, 0.57 nm and 0.99 nm, increasing about 0.33 nm, 0.28 nm and 0.49 nm with each step, for directions [001], [1$\bar{1}$1] and [1$\bar{1}$0], respectively. The discrete step size in each direction is given by the Nb(110) surface lattice and the energetically favored adsorption site of the adatoms being the hollow site between the Nb surface atoms.

Supplementary Note 6: Oscillations in peak intensities as a function of adatoms separation in the superconducting regime

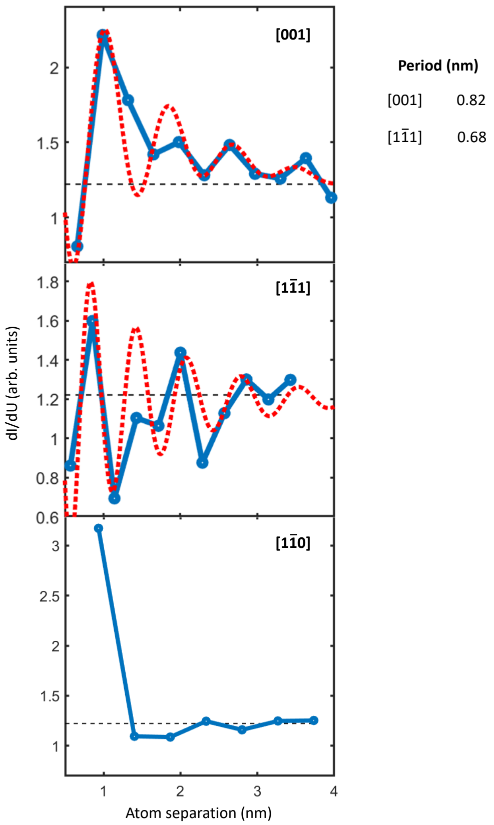

Supplementary Fig. 6: Fitting (dashed red curves) of the oscillating behaviour observed for hybridized YSR states as a function of the distance between the two adatoms along three distinct crystallographic directions, i.e. [001], [1$\bar{1}$1], and [1$\bar{1}$0]. The interaction is rapidly damped along the [1$\bar{1}$0] direction, severely complicating the analysis on any oscillating behavior. A much longer interaction is detected along [001], [1$\bar{1}$1], where a clear oscillating behaviour is visible. The wavelength of the oscillations can be extracted by fitting the data, providing the following values: 0.82 nm for [001], and 0.68 nm for [1$\bar{1}$1], respectively. The highly anisotropic behaviour can be traced back to the anisotropy of the Fermi surface. Good nesting vectors between flat parts of the Fermi surface can have a focusing effect on the wave function of quasi particles, significantly enhancing the coupling strength over larger distances. However, the discrete sampling induced by the lattice can lead to the aliasing effect between the periodicity of the lattice and the wavelength of the long-range oscillations, which complicates the identification of a specific region of the Fermi surface responsible for the oscillatory behavior. This is possible only in the asymptotic limit, i.e. at very large distances beyond those tracked experimentally, where the constructive interferences emanating from the nesting regions prevail. This is a well-known problem first highlighted within the context of indirectly coupled magnetic multilayers as discussed in Ref.[1].

Supplementary Note 7: Distance-dependence interaction effects in the metallic regime

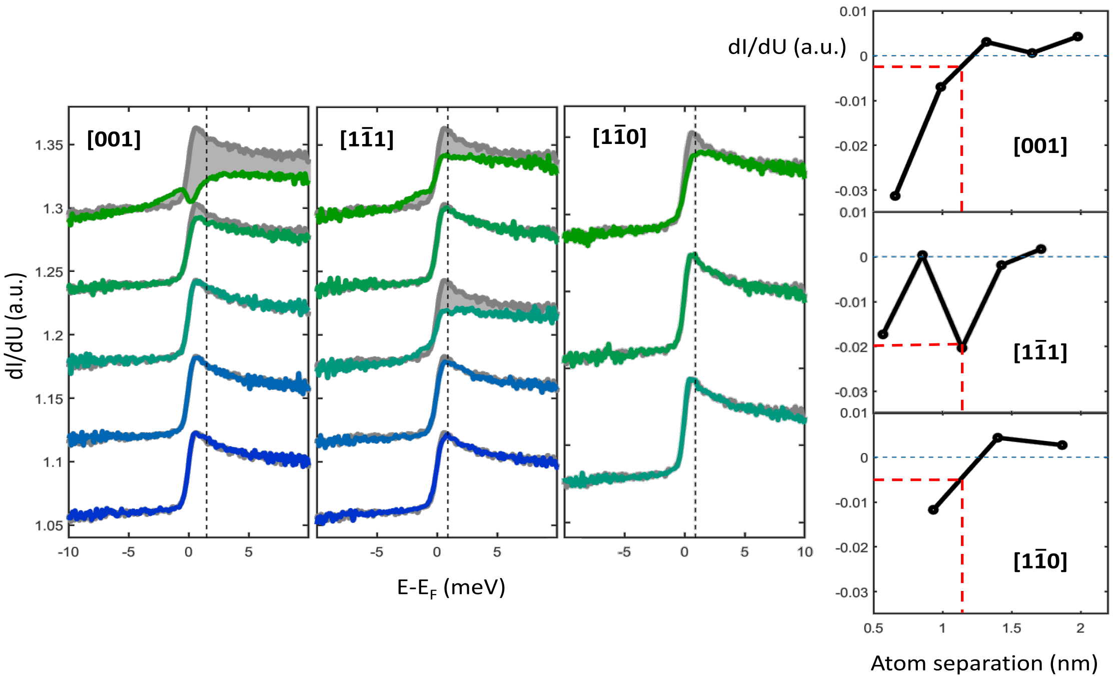

Supplementary Fig. 7: Differences induced by an additional adatom (color line) with respect to the single adatom case corresponds (gray line) for the three distinct crystallographic directions: i.e. [001], [1$\bar{1}$1] and [1$\bar{1}$0]. Three panels to the left show the STS data taken on one atom of the dimer with increasing atom separation from top to bottom and for the three crystallographic directions [001], [1$\bar{1}$1] and [1$\bar{1}$0]. The black dashed line indicates the energy at which the difference was measured and plotted in the panel to the right as a function of atom separation. As highlighted by the red dashed, the effect is longer range along the [1$\bar{1}$1] direction, being the change induced by an additional adatom at a distance of 1.14 nm, approximately eight and four times stronger than those observed along [001] and [1$\bar{1}$0] directions, respectively. Along all directions, the difference becomes very small from the 4th step onward.

Supplementary Note 8: Magnetic exchange interactions and ground states

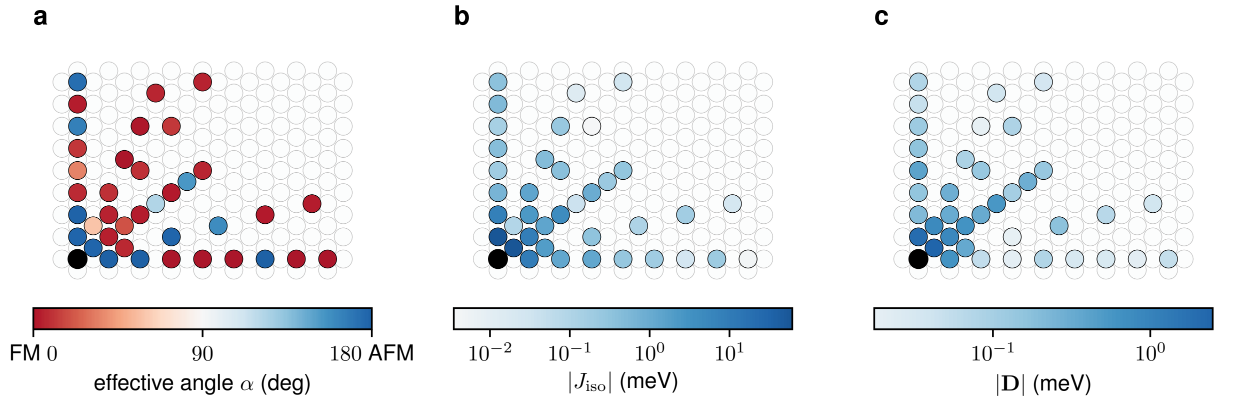

Supplementary Fig. 8: Magnetic properties of the different Cr dimers from first-principles. Each circle represents a dimer composed out of a Cr adatom at the respective position and the central Cr atom (black circle in the lower left corner). **a** Effective angle between the two dimer atoms. 0° corresponds to a ferromagnetic alignment and 180° to an antiferromagnetic alignment. **b** Isotropic exchange interaction J in units of [meV]. **c** Dzyaloshinskii-Moriya interaction $|\mathbf{D}|$ in units of [meV]. Note that the interactions are shown on a logarithmic scale.

The magnetic structure is obtained by minimizing the generalized Heisenberg model

$$\mathcal{H} = \sum_i \mathbf{e}_i \mathcal{K}_i \mathbf{e}_i + \frac{1}{2}\sum_{ij} J_{ij}\mathbf{e}_i \cdot \mathbf{e}_j + \frac{1}{2}\sum_{ij} \mathbf{D}_{ij} \cdot (\mathbf{e}_i \times \mathbf{e}_j) \quad , \tag{1}$$

containing the magnetic on-site anisotropy $\mathcal{K}_i$ of atom i, the isotropic exchange interaction J_{ij} and the Dzyaloshinskii-Moriya interaction $\mathbf{D}_{ij}$. After performing ab-initio calculations, the pair interactions were calculated using the method of infinitesimal rotations [2, 3]. In general, the magnetic coupling is weak, except for the nearest-neighbor dimers, which are not part of the experimental study. Therefore, we approximated the on-site anisotropy of each dimer atom by the one of an isolated Cr adatom, which was obtained using the method of constraining fields [4]. The effective angles $\alpha_{ij} = \arccos(\mathbf{e}_i \cdot \mathbf{e}_j)$ formed by the two dimer atoms are shown in Supplementary Figure 8a. The isotropic exchange and the Dzyaloshinskii-Moriya interaction strength are shown in Supplementary Figure 8b and c, respectively. The on-site anisotropy is given by

$$\mathcal{K} = \begin{pmatrix} -0.02 & 0.00 & 0.00 \\ 0.00 & -0.17 & 0.00 \\ 0.00 & 0.00 & 0.19 \end{pmatrix} \text{meV} \quad . \tag{2}$$

Supplementary Note 9: Calculation of the YSR energies

Using the inverse of the Green function of the dimer obtained from first-principles, we construct the hybridization function of the dimer complexes

$$\mathcal{H} = E - G^{-1}(E) \quad , \tag{3}$$

which contains various information such as the hybridization strength with the surface, the crystal field splitting, the strength of spin-orbit coupling, and the strength of the direct hopping between the dimer atoms. The hamiltonian can be written as an effective tight-binding model, where the on-site part is given by (omitting the atom index i on the parameters)

$$\begin{aligned}\mathcal{H}_i = \sum_{mm'}\sum_{ss'}\big(&E_d\,\delta_{mm'}\delta_{ss'} + U\,\mathbf{e}\cdot\boldsymbol{\sigma}_{ss'}\delta_{mm'} + \lambda\,\mathbf{L}_{mm'}\cdot\boldsymbol{\sigma}_{ss'} + \Delta^{(\mathrm{re})}_{mm'}\delta_{ss'} \\ &+ \mathrm{i}\Gamma\,\delta_{mm'}\delta_{ss'} + \mathrm{i}\Delta^{(\mathrm{im})}_{mm'}\delta_{ss'}\big)c^{\dagger}_{ims}c_{im's'} \quad ,\end{aligned} \tag{4}$$

and the inter-atomic hopping part is given by

$$\mathcal{H}_{ij} = \sum_{mm'}\sum_{s} t_{mm'}c^{\dagger}_{ims}c_{jm's} \quad . \tag{5}$$

E_d is the average energy of the d-orbitals with respect to the Fermi energy, $2U$ represents the exchange splitting of the magnetic moment pointing along $\mathbf{e}$, $\boldsymbol{\sigma} = (\sigma_x, \sigma_y, \sigma_z)$ is the vector of Pauli matrices, λ is the strength of the local spin-orbit coupling, $\mathbf{L}$ is the local orbital angular momentum operator, $\Delta^{(\mathrm{re})}$ is an orbital dependent energy shift corresponding to the crystal field splitting, Γ and $\Delta^{(\mathrm{im})}$ are non-hermitian contributions that result from the hybridization with the substrate, and $t_{mm'}$ is the orbital-dependent hopping between atoms i and j.

Using the scheme described in the Supplementary Information of Ref. [5] the effective non-magnetic and magnetic scattering contributions, V_m and J_m, of the impurity-substrate s-d interaction $\mathcal{I}_m$ can be obtained by virtue of the Schrieffer-Wolff transformation [6], which gives access to the energies of the YSR states [7, 8],

$$\frac{\epsilon_m}{\Delta} = \pm\frac{1 + {\beta_m}^2 - {\alpha_m}^2}{\sqrt{[1 + {\beta_m}^2 - {\alpha_m}^2]^2 + 4{\alpha_m}^2}} \quad . \tag{6}$$

The parameters α_m and β_m can be directly obtained from ab-initio by diagonalizing the effective Hamiltonian construction given in eqs. (4) and (5),

$$\beta_m = \pi\rho V_m = -\frac{\Gamma_m E_m}{(E_m + U)(E_m - U)}; \quad \alpha_m = \pi\rho J_m S = \frac{\Gamma_m U}{(E_m + U)(E_m - U)} \quad . \tag{7}$$

Note that this scheme is based on the eigenstates of a single atom. To account for the impact of the hopping t_{ij} between the two dimer atoms, we repeat the effective Hamiltonian construction down to the single atom. Using this procedure the local on-site parameters are renormalized by the hopping, which allows to quantify the impact of the hopping on the YSR energies. The bare parametrization obtained for the isolated Cr adatom ($\alpha_{d_{z^2}} = 0.795$ and $\beta_{d_{z^2}} = -0.140$) can be found in the Supplement of Ref. [5] .

The change of the YSR energies of the experimentally relevant orbital with z^2 character ($m = 0$) is shown in Supplementary Figure **9**a using the realistic structure given in Supplementary Figure **8**a. To account for the impact of the magnetic structure Supplementary Figure **9**b and c shows the shift of the YSR energies for ferromagnetic and antiferromagnetic dimers, respectively. Most affected by the magnetic configuration are the next-nearest neighbor dimers in the [001] and [$1\bar{1}0$] directions. After a systematic comparison with the experimental data, we noted that one of the dimers along [$1\bar{1}0$] were better described with a ferromagnetic coupling instead of the antiferromagnetic coupling. Since the magnetic exchange interaction of this particular dimer is of the order of 1 meV, which in addition undergoes a couple of uncertainties like the impact

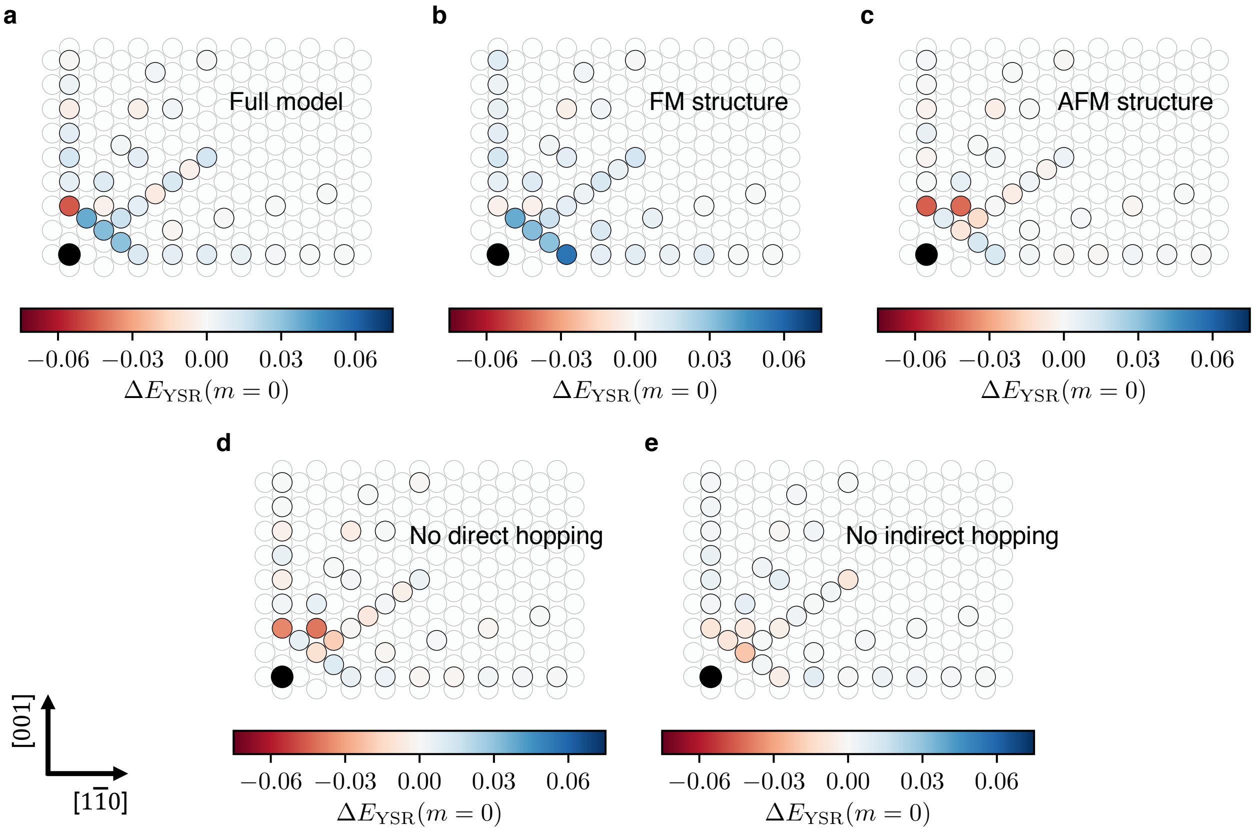

Supplementary Fig. 9: Shift of the YSR energies of the z^2-orbital of the Cr atoms based on various model assumption for all considered Cr dimers. The first three panels show the full tight-binding model with the realistic magnetic structure (**a**), as shown in Supplementary Figure 8, a ferromagnetic configuration for all dimers (**b**) and an antiferromagnetic configuration for all dimers (**c**). The impact of the coupling between the dimer atoms on the YSR energy is shown in the last two panels using the realistic magnetic structure. No direct hopping t_{ij} is considered (**d**) and additionally also no renormalization of the orbital-dependent broadening $\Delta_i^{(\mathrm{im})}$ is used, which can be seen as an indirect hopping effect (**e**).

of the atomic relaxations or a renormalization due to zero-point quantum fluctuations [9], we used for that particular dimer in Figure 3 of the main manuscript the result obtained with the ferromagnetic coupling instead of the antiferromagnetic one.

Supplementary Figure 9d and e show the impact of the direct hopping t_{ij} and the orbital-dependent hybridization $\Delta^{(\mathrm{im})}$ using the realistic magnetic structure. For most dimers the direct hopping has a crucial contribution to the shift of the YSR energies. Only the dimer in the [001] direction shows still a significant shift without the direct hopping t_{ij}, which vanishes if in addition $\Delta^{(\mathrm{im})}$, which can be seen as an additional substrate-mediated effect of the coupling between the dimer atoms, is neglected (panel e). In conclusion, the main origin of the shifts in the YSR energies can be attributed to the nature of the magnetic coupling and the hopping between the dimer atoms.

To shed some more light on the former aspect, we use in the following the Alexander-Anderson model as described in Ref. [10], which is a two-site model with a single orbital per site and spin channel. The main idea is that the nature of the coupling influences the formation of bonding and anti-bonding states [11]. For a ferromagnetic coupling the majority (minority) spin channels of both atoms can hybridize leading to a double Lorentzian structure symmetrically shifted by $\pm t_{12}$, which in terms of our effective single-particle Hamiltonian can be viewed as an increased broadening $\Gamma_{\mathrm{renorm}} = \Gamma + \Delta\Gamma$ while the effective spin splitting

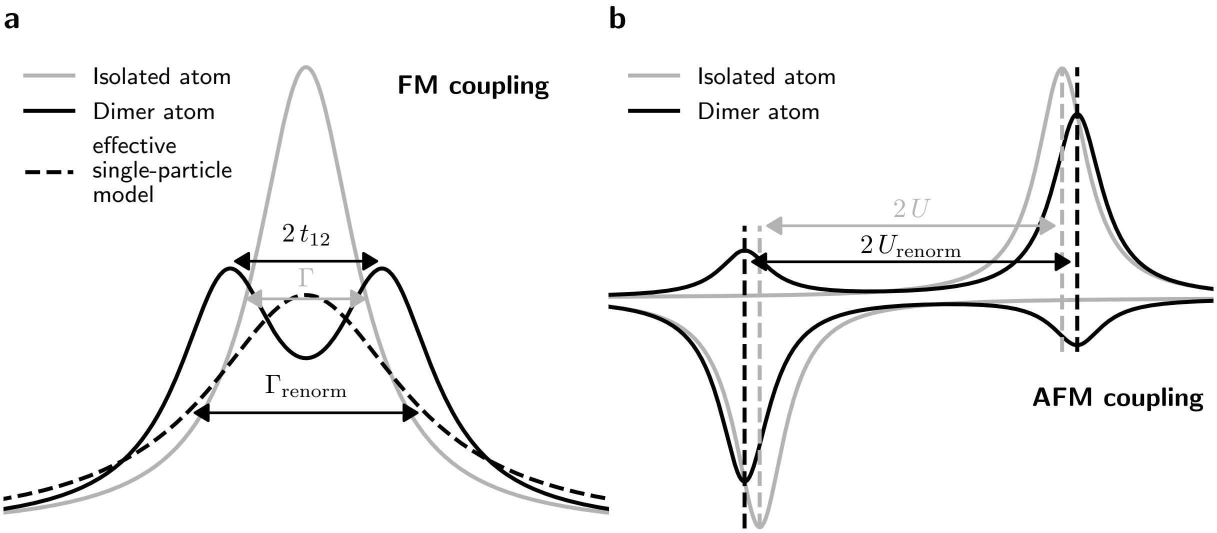

Supplementary Fig. 10: Illustration of the impact of ferromagnetic and antiferromagnetic coupling on the formation of bounding and anti-bounding states in a dimer. **a** Ferromagnetic coupling. A state of the isolated adatom (grey line) couples via the hopping t_{12} to the other dimer atom yielding a bonding and anti-bonding state (black line). In an effective single-particle single-orbital model (dashed line) it is reflected as an increased renormalized hybridization Γ_{renorm}. Only one spin channel is shown for the sake of simplicity. **b** Antiferrormagnetic coupling. The coupling between the dimer atoms reflects itself as an effective renormalized spin splitting U_{renorm}.

U remains unchanged (see illustration in Supplemenatry Figure **10**a), which can be shown to be given by $\Delta\Gamma \propto t^2/\Gamma$. In contrast, in the case of an anti-ferromagnetic coupling the majority spin channel of one atom couples to the minority spin channel of the other atom (and vice versa), which manifests itself in a shift of the energy levels and can be viewed as an increase of the effective spin splitting $U_{\mathrm{renorm}} = U + \Delta U$ and an unchanged broadening Γ in the single-particle description as illustrated in Supplementary Figure **10**b. The change in the effective spin splitting is given by $\Delta U = t^2/2U$ [10].

To analyze the impact of the magnetic coupling analytically, in the following we take the limit $E_m \ll U$ and $\Gamma \ll U$ for which eq. (6) simplifies to

$$\frac{\epsilon_m}{\Delta} = \pm\frac{1-{\alpha_m}^2}{1+{\alpha_m}^2} \quad , \tag{8}$$

with $\alpha_m = -\Gamma/U$. A change of Γ and U will modify α_m in the following fashion

$$\alpha_m + \Delta\alpha_m = -\frac{\Gamma}{U}\left(1 + \frac{\Delta\Gamma}{\Gamma} - \frac{\Delta U}{U}\right) \quad , \tag{9}$$

which yields

$$\frac{\epsilon_m}{\Delta} = \pm\frac{1-{\alpha_m}^2}{1+{\alpha_m}^2}\left(1 - \frac{2\alpha_m^3}{1-\alpha_m^4}\,\Delta\alpha_m\right) \quad . \tag{10}$$

Since for the FM and AFM coupling $\Delta\Gamma > 0$ and $\Delta U > 0$, respectively, we expect from eqs. (9) and (10) that FM and AFM coupling induce shifts of the YSR energies in opposite directions, which agrees well with our findings using the full model shown in Supplementary Figure **9**b and c.

Supplementary Note 10: Magnetic coupling

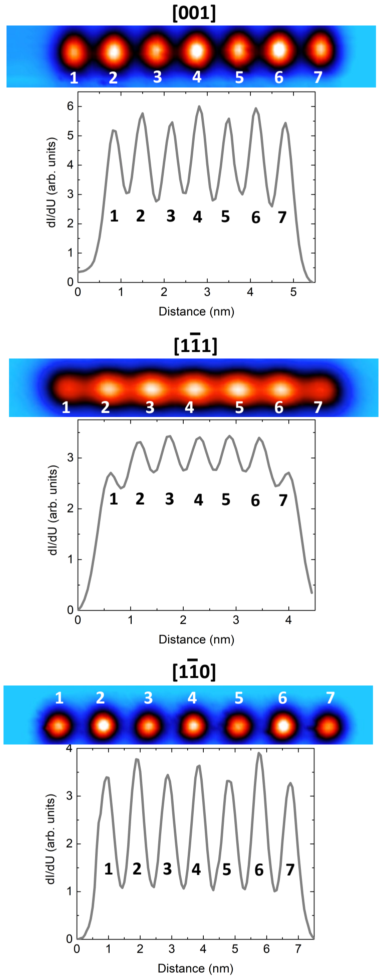

Supplementary Fig. 11: Differential conductance maps (dI/dU) and corresponding line profiles for chains assembled at next nearest neighbor distance along distinct crystallographic directions. For all chains, adatoms are positioned at the same interatomic distances considered for the dimers, i.e. next-nearest neighbours. The magnetic coupling at those distances do not change with the length of the chains. All data have been acquired with the very same Cr microtip. Set point parameters: V = 0.9 mV , I = 3 nA, lock-in modulation V = 200 μV (r.m.s.). To stabilize the magnetic structure against fluctuations, a magnetic field B = 1 T has been applied perpendicular to the sample surface. Both [001] and $[1\bar{1}0]$ directions are characterized by an alternating contrast, indicative of antiferromagnetic coupling between neighbouring Cr adatoms. Along the $[1\bar{1}1]$ direction, all adatoms inside the chain are characterized by the same contrast, indicating their ferromagnetic coupling. Small differences are visible for the adatoms at the end of the chains, due to their different local environment with respect to the adatoms inside the chains.

References

[1] Bruno, P. & Chappert, C. Ruderman-kittel theory of oscillatory interlayer exchange coupling. *Phys. Rev. B* **46**, 261–270 (1992). URL https://link.aps.org/doi/10.1103/PhysRevB.46.261.

[2] Liechtenstein, A. I., Katsnelson, M. I., Antropov, V. P. & Gubanov, V. A. Local spin density functional approach to the theory of exchange interactions in ferromagnetic metals and alloys. *Journal of Magnetism and Magnetic Materials* **67**, 65–74 (1987).

[3] Ebert, H. & Mankovsky, S. Anisotropic exchange coupling in diluted magnetic semiconductors: Ab initio spin-density functional theory. *Physical Review B* **79**, 045209 (2009).

[4] Brinker, S., Dias, M. d. S. & Lounis, S. The chiral biquadratic pair interaction. *New Journal of Physics* **21**, 083015 (2019).

[5] Küster, F. *et al.* Correlating Josephson supercurrents and Shiba states in quantum spins unconventionally coupled to superconductors. *Nature Communications* **12**, 1108 (2021).

[6] Schrieffer, J. R. & Wolff, P. A. Relation between the anderson and kondo hamiltonians. *Phys. Rev.* **149**, 491–492 (1966).

[7] Rusinov, A. I. On the Theory of Gapless Superconductivity in Alloys Containing Paramagnetic Impurities. *JETP* **29**, 1101 (1969).

[8] Balatsky, A. V., Vekhter, I. & Zhu, J.-X. Impurity-induced states in conventional and unconventional superconductors. *Rev. Mod. Phys.* **78**, 373–433 (2006).

[9] Bouaziz, J., Ibañez Azpiroz, J., Guimarães, F. S. M. & Lounis, S. Zero-point magnetic exchange interactions. *Phys. Rev. Research* **2**, 043357 (2020).

[10] Oswald, A., Zeller, R., Braspenning, P. J. & Dederichs, P. H. Interaction of magnetic impurities in Cu and Ag. *Journal of Physics F: Metal Physics* **15**, 193–212 (1985).

[11] Mavropoulos, P., Lounis, S. & Blügel, S. Exchange coupling in transition-metal nanoclusters on cu(001) and cu(111) surfaces. *Physica Status Solidi (b)* **247**, 1187–1196 (2010). URL https://onlinelibrary.wiley.com/doi/abs/10.1002/pssb.200945535.
